# Is intake of fruit juice useful in exercise-induced hypoglycemia prevention in individuals with type 1 diabetes mellitus?

**DOI:** 10.3389/fendo.2022.1045639

**Published:** 2022-10-21

**Authors:** Sarah Valder, Christian Brinkmann

**Affiliations:** ^1^ Institute of Cardiovascular Research and Sport Medicine, Department of Molecular and Cellular Sport Medicine, German Sport University Cologne, Cologne, Germany; ^2^ Institute of Cardiovascular Research and Sport Medicine, Department of Preventive and Rehabilitative Sport Medicine, German Sport University Cologne, Cologne, Germany; ^3^ IST University of Applied Sciences, Düsseldorf, Germany

**Keywords:** type 1 diabetes mellitus, exercise, hypoglycemia, fruit juice, fructose, glucose

## Introduction

Aerobic exercise normally leads to an increase in skeletal muscle glucose uptake (due to widely insulin-independent contraction-induced skeletal muscle glucose uptake, increase in skeletal muscle insulin sensitivity and blood flow), a decrease in endogenous insulin secretion and an increase in hepatic glucose production in the liver to maintain euglycemia (1, 2). This exercise-associated glucose homeostasis in healthy individuals results from a complex endocrine interaction between insulin as a blood glucose lowering hormone and the main counter-regulatory hormones glucagon, catecholamines, cortisol, and growth hormone (3). In individuals with type 1 diabetes mellitus (T1DM), the mentioned hormonal balance, especially between insulin and glucagon, is disturbed. One problem is that injected insulin can further increase glucose uptake in skeletal muscle and may block glucagon-mediated hepatic glucose production resulting in exercise-induced hypoglycemia (3, 4). Hypoglycemia may still occur in the post-exercise phase, as increased skeletal muscle insulin sensitivity and glucose uptake can last for up to 48 hours following acute exercise (5). Hypoglycemia is something many persons with T1DM are concerned about, which is one of the reasons why many of them avoid sports and exercise (1, 6). However, the health benefits of regular exercise are particularly important for individuals with T1DM to reduce the risk and progression of secondary complications (7).

It is recommended for physically active persons with T1DM to monitor their glucose levels before, during and after exercise (1). In addition, they should be sensitized to adjust their insulin dose and/or carbohydrate (CHO) intake to different types, durations and intensities of exercise to maintain stable and safe blood glucose levels (4). In their excellent expert position statement, Moser et al. (8) recommend suitable strategies for individuals with T1DM based on their glucose values and glucose trends, measured or calculated using continuous glucose monitoring (CGM) devices.

When T1DM persons spontaneously decide to exercise, their only option is usually to increase carbohydrate intake. For low- to moderate-intensity aerobic activities (duration 30 – 60 min), a pre-exercise intake of approx. 15 g CHOs may prevent hypoglycemia during exercise (4, 9). For more prolonged exercise sessions (> 60 min), the recommended CHO amount increases up to 30 – 90 g to stabilize blood glucose levels (1).

To promote long-term health, individuals with T1DM should be instructed to choose nutrient-dense CHO foods (4, 10). Fruit juice seems to be an advantageous food source because it can be easily consumed and has a relatively high nutritional density rich in vitamins, carotenoids and polyphenols (11, 12).

The consumption of fruit juice is generally perceived as controversial due to its relatively high sugar content (e.g., glucose, fructose and sucrose) and a relatively low content of fibers (11). This opinion article sheds light on the current state of knowledge on the intake of fruit juice to prevent exercise-induced hypoglycemia in individuals with T1DM. Furthermore, other health-related issues associated with fruit juice intake in the context of physical activity will be discussed and directions for future research will be identified.

## Fruit juice intake for exercise-induced hypoglycemia prevention

The effectiveness of various CHO sources and dosage forms in the treatment of hypoglycemia (including fruit juice) has already been studied (13–17). According to the American Diabetes Association’s (ADA) recommendations, individuals with T1DM should choose rapidly-acting glucose to treat severe hypoglycemia (18). Recently published data show that fruit juice was the most frequently chosen option among persons with T1DM in hypoglycemic events (70%) (19).

A previous study investigated the response to three types of frequently used rapidly-acting CHOs (defined glucose solution (glycemic index (GI) = 100) vs. sugar candies (GI = 70) vs. fruit juice (GI = 57)) in adolescents with T1DM who participated in a trekking camp for 5 days (20). All three treatments were similar in regulating exercise-induced hypoglycemia. There were no statistically significant differences between the three treatments in terms of duration of hypoglycemia and the frequency of hypoglycemic events after hypoglycemia correction (20).

To our knowledge, no studies have been conducted on pre-exercise fruit juice intake for the prevention of exercise-induced hypoglycemia in persons with T1DM. The question arises whether fruit juice intake is similar or might even be more effective than other CHO ingestion strategies in exercise-induced hypoglycemia prevention.

**Figure 1:**
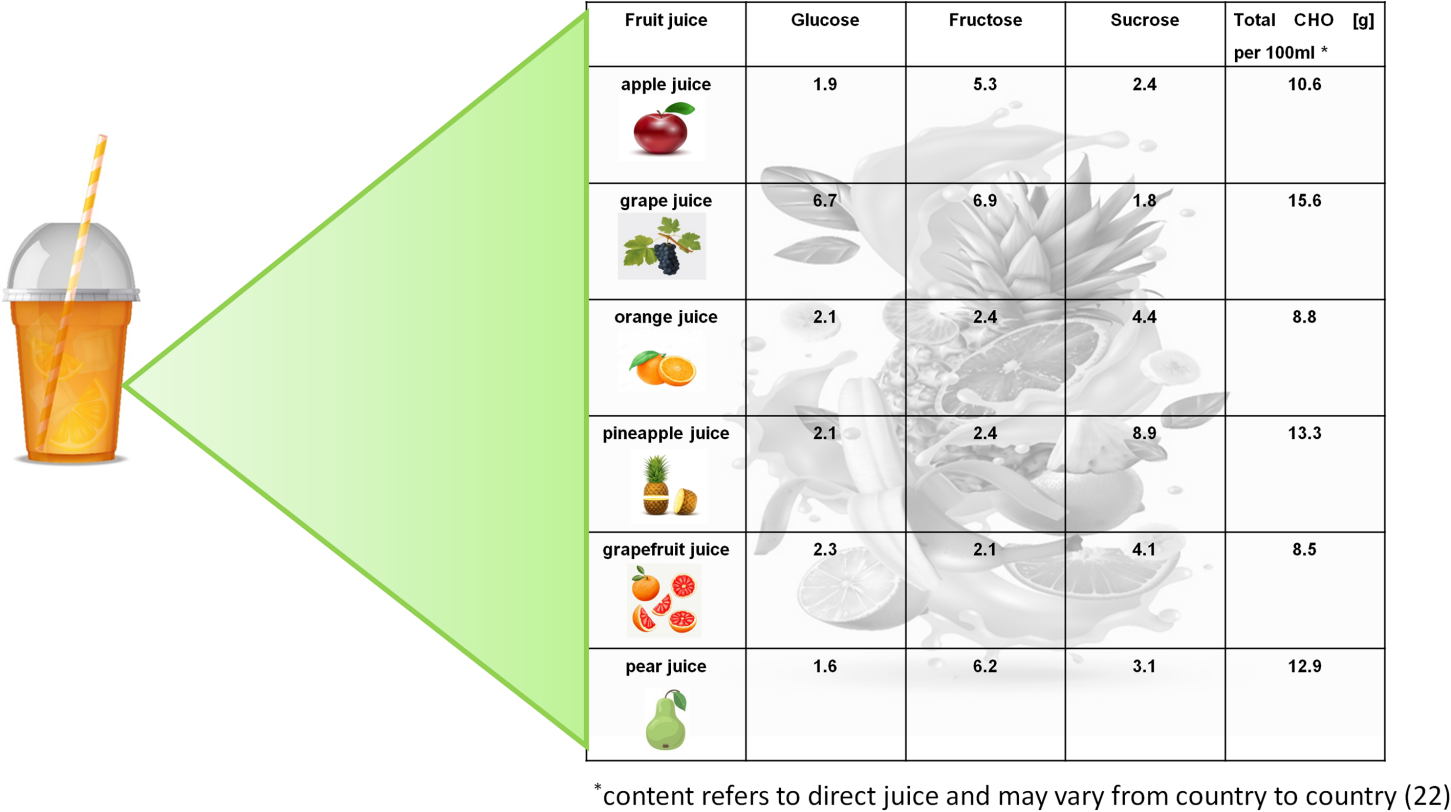
Glucose, fructose and sucrose contents [g/100 ml] in different fruit juices. Graphics: https://freepik.com.

Bally et al. (6) investigated glycemic and other metabolic effects of glucose-fructose co-ingestion (among other CHOs, this CHO composition (glucose+fructose) is contained in many juices, **Figure 1**) with glucose-only ingestion to maintain glycemia in physically active individuals with T1DM who participated in two 90-min iso-energetic continuous cycling sessions at 50% VO_2max_ (without insulin adjustment). Concentrations of blood glucose and insulin were comparable between treatments (recorded up to 2 h post-exercise). This study found significantly higher fat oxidation and lower CHO oxidation with a muscle glycogen-sparing effect under the glucose-fructose co-ingestion treatment compared with the glucose-only treatment.

Kosinski et al. (2) recently conducted a pilot study that investigated whether pre-exercise intake of fructose decreases the risk of exercise-induced hypoglycemia in individuals with T1DM. Male adults with T1DM underwent two 60-min aerobic cycling sessions at 50% maximal power with prior intake (30 min pre-exercise) of 20 g fructose dissolved in water versus water only. Exercise was performed in the morning in the fasted state without insulin adjustment. Time to hypoglycemia was the primary outcome, supervised using a continuous glucose monitoring system. Pre-exercise fructose intake resulted in an 88% decreased risk of exercise-induced appearance of hypoglycemia with significantly higher glucose levels compared to the control situation. Fat oxidation rates between the two test conditions were comparable, with suppressed fat oxidation in the fructose ingestion treatment 30 min post-exercise.

Based on the results of Bally et al. and Kosinski et al. (2, 6), it can be deduced that fruit juice can be used to prevent exercise-induced hypoglycemia in individuals with T1DM. Fat oxidation may also remain more effective following glucose-fructose-sucrose co-ingestion (when drinking fruit juice) compared with ingesting glucose only.

## Other health-related aspects of fruit juice intake in the context of physical activity

### Fructose malabsorption

Fructose intake from fruit juice has been intensively discussed because of the side effects related to possible fructose malabsorption (10, 21). Symptoms of fructose malabsorption can arise when fructose is fermented by bacteria in the distal small intestine and colonic lumen. This fermentation process can lead to rapid accumulation of a mixture of gases. Together with the osmotic action exerted by malabsorbed fructose in the intestinal lumen, water influx and subsequent retention in the lumen of the small and large intestine can be increased, resulting in symptoms such as abdominal pain, bloating or diarrhea (21). There is no clear evidence yet which fruit juices are recommendable for pre-exercise intake to prevent hypoglycemic events in individuals with T1DM without causing discomfort. However, fructose-sensitive persons should choose fruit juices that contain a glucose to fructose ratio of approximately 1:1, such as in grape, orange, pineapple or grapefruit juice (22). The equivalent amount of glucose in these juices is likely to increase the tolerability of fructose and might even prevent potential gastrointestinal side effects (23). To date, the physiological mechanisms behind this phenomenon have not yet been fully understood. There is some evidence that the brush-border disaccharidase sucrase-isomaltase and its related transport system that can cleave sucrose and transport the resultant monomers (glucose and fructose), can also co-transport glucose and fructose when co-ingested as monosaccharides (23–25).

### Antioxidative properties

Additionally, more research work should focus on the antioxidative effects of fruit juices. Vitamins or polyphenols can be potent mediators of antioxidative effects (12). Free radical production is increased through exercise which can contribute to muscle damage and impaired immunity (26). An interesting question is whether pre-exercise fruit juice intake can decrease exercise-associated free radical availability, thereby supporting regeneration in physically active persons with T1DM. A study involving healthy volunteers has shown that the intake of smoothies can accelerate recovery from exercise-induced muscle damage (27). On the other hand, it cannot be excluded that the intake of fruit juice with antioxidative properties shortly before acute exercise can attenuate some exercise-induced adaptation processes because free radicals also act as signaling molecules, activating redox-sensitive molecular pathways that are relevant for important beneficial training adaptations (28).

### Daily fruit intake recommendations

Recently published data show that people who drink fruit juice are more likely to achieve the recommended “5 a day rule” for fruit and vegetable consumption than those who avoid fruit juice intake (11). There is strong evidence that fruit and vegetable consumption is associated with a reduced rate of all-cause mortality and disease risks (29). Therefore, the intake of fruit juice could be useful in the context of a healthy lifestyle based on regular exercise and a balanced diet.

## Conclusions and recommendations for future research

Many individuals with T1DM use fruit juices to prevent (exercise-induced) hypoglycemia in their daily exercise routines. It has been demonstrated that glucose-fructose co-ingestion can prevent exercise-induced hypoglycemia in persons with T1DM. While direct scientific evidence for the use of fruit juice intake to prevent exercise-induced hypoglycemia in individuals with T1DM is still lacking, it is, in our opinion, very likely that this strategy is effective. We recommend appropriately dosing juices to provide sufficient carbohydrates to meet the increased glucose requirement during exercise to stabilize T1DM individuals’ blood glucose levels. However, we also suggest taking other health-related aspects into consideration that go beyond the mere intention of using juices to prevent exercise-induced hypoglycemia. Regarding fructose malabsorption, the tolerability of different fruit juices should be individually tested before the first intake prior to physical activity. The extent of any further health benefits and whether the intake of fruit juices due to the antioxidative properties of their components can indeed reduce recovery times in ambitious athletes should be clarified in further studies. Whether the antioxidative properties could attenuate positive adaptation processes also needs to be clarified in future studies.

## Author contributions

Conceptualization, CB. Literature research, SV. Writing—original draft preparation, SV. Writing—review and editing, CB and SV. Visualization, SV. Supervision, CB. All authors contributed to the article and approved the submitted version.

## Conflict of interest

Author CB is a member of the Abbott Advisory Board. Abbott had no role in manuscript preparation or decision to publish.

The remaining author declares that the research was conducted in the absence of any commercial or financial relationships that could be construed as a potential conflict of interest.

## Publisher’s note

All claims expressed in this article are solely those of the authors and do not necessarily represent those of their affiliated organizations, or those of the publisher, the editors and the reviewers. Any product that may be evaluated in this article, or claim that may be made by its manufacturer, is not guaranteed or endorsed by the publisher.
